# Identification of the promising olive (*Olea europaea* L.) cultivars based on morphological and pomological characters

**DOI:** 10.1002/fsn3.2767

**Published:** 2022-02-07

**Authors:** Ali Khadivi, Farhad Mirheidari, Younes Moradi, Simin Paryan

**Affiliations:** ^1^ Department of Horticultural Sciences, Faculty of Agriculture and Natural Resources Arak University Arak Iran

**Keywords:** breeding, fruit, morphology, olive, yield

## Abstract

Olive (*Olea europaea* L.) is an ancient tree and can tolerate drought very well. In the present study, morphological and pomological diversity of 24 olive cultivars (5–15 replications for each cultivar, 243 trees in total) was evaluated. There were significant differences among the cultivars studied based on the characters recorded. The CV was more than 20.00% in 46 of 50 characters measured. Leaf length ranged from 27.07 to 78.54 mm, and leaf width varied from 5.42 to 23.06 mm. Ripening date ranged from late‐August to early‐October. Fruit length ranged from 13.04 to 33.72 mm, fruit diameter varied from 10.24 to 23.71 mm, fruit weighted from 0.97 to 9.61 g, and the range of fruit flesh thickness was 1.63–7.65 mm. There was high variability in terms of fruit color, ranging from light green to black. Hierarchical cluster analysis (HCA) performed based on the mean of replications with Euclidean distance and Ward method grouped the cultivars into two major clusters. Differences in many of the morphological traits were observed across the cultivars. These sets of data were used to identify unique and desirable cultivars morphologically. The present research demonstrates that local olive cultivars have unique characteristics that differentiate them from imported cultivars. Thus, local cultivars provide novel genetic resources that should be conserved.

## Graphical abstract

The present research demonstrates that local olive cultivars have unique characteristics that differentiate them from imported cultivars. These sets of data were used to identify unique and desirable cultivars morphologically. Thus, local cultivars provide novel genetic resources that should be conserved.

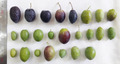

## 1 INTRODUCTION

Olive (*Olea europaea* L.) belongs to a dicotyledonous family *Oleaceae*. It is an ancient tree which has been found in Egyptian tombs from 2000 years BC. Olive tree of Vouves is considered as the oldest olive tree in the world and it is estimated to be over 3000 years old (Maravelakis et al., 2012). It is found in all regions of the world except arctic. However, 98% of the world olive cultivation is carried out in Mediterranean region, and it contributes a major share in olive oil production (Hashmi et al., 2015). More than 2000 olive cultivars are present in Mediterranean basin and these cultivars are characteristically distinguished through tree and fruit morphology (Bartolini et al., 1998; Ganino et al., 2006).

Botanically, olive is an evergreen tree of subtropical nature. It can attain the height of up to 10 m or more. Leaves are shortly stalked, oblong or lanceolate in their shape. White creamy flowers are produced in leaf axils. Fruit is drupe, ovoid in shape, and blackish‐violet in color when ripe (Shu, 1996). It is a monoecious plant and pollination occurs through wind. Genetically, it possesses a diploid set of chromosomes as 2*n* = 46 (Kumar et al., 2011). Olive thrives well in climates having hot summers with low humidity and cold winters. Winter chilling of at least 2 months is required for flower bud initiation. However, it cannot withstand freezing temperature, which ultimately leads to death of the plant. It can tolerate drought very well and can be successfully grown in areas with annual rainfall of 900–1000 mm. It can withstand moderate soil conditions, but water logging conditions are injurious for plant health (Munir, 2009).

The cross‐pollinating nature of olive and its secular history contributed to determine a wide germplasm biodiversity with a large number of more than 1200 cultivars present in the main olive oil producing countries (Bartolini et al., 2005). This genetic diversity could be an important resource for the development of modern olive culture toward typical olive oil and fresh productions. This richness in terms of available biodiversity, however, often has determined some drawbacks in the management and identification of the plant material to distinguish between cultivars, and this has been further complicated by the frequency of homonyms and synonyms (Hegazi et al., 2012).

Morphological and agronomic characters have been widely used for descriptive purposes (Khadivi & Arab, 2021; Khadivi et al., 2021; Mirmahdi & Khadivi, 2021) and are commonly used to distinguish olive cultivars (Arias‐Calderon et al., 2014; Barranco et al., 2000; Rotondi et al., 2003, 2011; Trentacoste & Puertas, 2011). Biometric indexes should always be accompanied by a detailed morphological description of the organs (inflorescence, leaf, fruit, and stone) of olive varieties following the UPOV method (Barranco et al., 2000). Many researchers observed that different cultivars are morphologically variable based on geographical locations and under various plant growth management practices (Grati et al., 2002; Youssefi et al., 2011).

The present research aimed to investigate the phenotypic characterizations of olive cultivars from Gilvan area in Zanjan province/Iran.

## 2 MATERIALS AND METHODS

### 2.1 Plant material

Morphological and pomological diversity of 24 olive cultivars (5–15 replications for each cultivar, 243 trees in total) was evaluated at a collection in Gilvan area in Zanjan province/Iran. Gilvan area is located at 36º44′20′′N latitude, 48º53′42′′E longitude, and 1080 m height above sea level. The cultivars were between 10 and 12 years old and were healthy and in full fruiting stage. The orchard management operations, including nutrition, irrigation, and pest and disease control, were performed regularly and uniformly for the cultivars.

### 2.2 The characters evaluated

Fifty morphological and pomological traits were used to evaluate phenotypic diversity (Table 1). A total of 50 adult leaves and 50 mature fruits per cultivar were randomly selected and harvested. The traits related to dimensions of leaf, fruit, and stone were measured using a digital caliper. A digital scale with an accuracy of 0.01 g was used to measure the weight of fruit and stone. The qualitative traits (Table 2) were visually examined and coded according to the olive descriptor (UPOV, Barranco et al., 2000).

**TABLE 1:** Statistical descriptive parameters for morphological traits used to study olive cultivars

No.	Character	Abbreviation	Unit	Min.	Max.	Mean	*SD*	CV (%)
1	Tree growth habit	TGH	Code	1	5	2.77	1.53	55.05
2	Tree growth vigor	TGV	Code	1	5	3.91	1.11	28.31
3	Tree height	TH	Code	1	5	3.36	1.39	41.22
4	Trunk diameter	TD	Code	1	5	3.27	1.40	42.81
5	Trunk color	TC	Code	1	5	2.34	1.42	60.60
6	Canopy density	CADe	Code	1	5	4.12	1.08	26.09
7	Branching	B	Code	1	5	3.81	1.11	29.16
8	Branch density	BDe	Code	3	5	4.14	0.99	23.99
9	Branch flexibility	BF	Code	1	5	4.19	1.16	27.57
10	Skin color of perennial branch	SCPB	Code	1	7	5.02	2.10	41.77
11	Skin color of current branch	SCCuB	Code	1	5	2.92	0.77	26.27
12	Leaf density	LDe	Code	3	5	4.44	0.90	20.27
13	Leaf length	LLe	mm	27.07	78.54	51.41	10.80	21.01
14	Leaf width	LWi	mm	5.42	23.06	10.59	2.82	26.67
15	Petiole length	PeLe	mm	1.79	9.56	4.90	1.29	26.28
16	Petiole diameter	PeD	mm	0.50	1.53	0.92	0.14	15.28
17	Leaf upper surface color	LUSuC	Code	1	5	3.32	1.18	35.66
18	Transparency of leaf upper color	TrLUC	Code	1	5	4.17	1.20	28.75
19	Leaf lower surface color	LLoSuC	Code	1	3	1.51	0.87	57.88
20	Leaf shape	LSh	Code	1	7	4.87	1.32	27.04
21	Leaf apex shape	LASh	Code	1	5	3.86	1.36	35.16
22	Leaf base shape	LBsSh	Code	1	3	1.55	0.90	57.81
23	Ripening date	RiDa	Date	Late‐Aug	Early‐Oct	5.51	2.49	45.26
24	Fruit density	FrDe	Code	1	5	3.25	1.48	45.51
25	Mean of fruit number in inflorescence	MFNo	Number	1	10	3.32	2.29	68.92
26	Fruit stalk length	FrStLe	mm	1.04	10.93	3.93	1.82	46.31
27	Fruit stalk diameter	FrStD	mm	0.54	2.07	0.98	0.23	23.78
28	Fruit shape	FrSh	Code	1	5	3.39	1.45	42.89
29	Fruit Symmetry	FrSy	Code	1	5	2.88	1.60	55.42
30	Fruit apex shape	FrASh	Code	1	3	1.86	0.99	53.39
31	Fruit base shape	FrBsSh	Code	1	3	1.77	0.98	55.14
32	Fruit nipple shape	FrNiSh	Code	1	5	2.37	1.50	63.33
33	Fruit length	FrLe	mm	13.04	33.72	22.52	4.09	18.16
34	Fruit diameter	FrWi	mm	10.24	23.71	15.77	3.25	20.58
35	Fruit color	FrC	Code	1	15	7.44	4.93	66.24
36	Lenticel on fruit skin	LenFrSk	Code	1	5	3.91	1.61	41.10
37	Fruit weight	FrWe	g	0.97	9.61	3.44	1.81	52.44
38	Fruit flesh firmness	FrFlFi	Code	1	5	3.86	1.33	34.56
39	Fruit flesh thickness	FrFlTh	mm	1.63	7.65	4.03	1.14	28.36
40	Stone shape	StSh	Code	1	7	4.03	1.73	42.88
41	Stone Symmetry	StSy	Code	1	5	2.65	1.45	54.60
42	Stone apex shape	StASh	Code	1	3	1.40	0.80	57.00
43	Stone base shape	StBsSh	Code	1	5	3.27	1.51	46.27
44	Stone length	StLe	mm	9.13	25.71	16.35	3.18	19.47
45	Stone diameter	StD	mm	4.82	12.73	8.22	1.59	19.40
46	Stone color	StC	Code	1	5	3.26	1.34	41.20
47	Stone surface	StSu	Code	1	5	2.99	1.45	48.46
48	Groove number on stone	GNoSt	Code	1	5	2.30	1.05	45.83
49	Stone weight	StWe	g	0.20	1.79	0.70	0.34	47.74
50	Flesh ratio to stone	FlSt	Ratio	1.02	7.68	3.91	1.22	31.16

**TABLE 2:** Frequency distribution for the measured qualitative morphological characters in the studied olive cultivars

Character	Frequency (no. of cultivars)							
	1	3	5	7	9	11	13	15
Tree growth habit	Drooping (86)	Spreading (99)	Erect (58)	–	–	–	–	–
Tree growth vigor	Low (7)	Moderate (119)	High (117)	–	–	–	–	–
Tree height	Low (40)	Moderate (119)	High (84)	–	–	–	–	–
Trunk diameter	Low (45)	Moderate (120)	High (78)	–	–	–	–	–
Trunk color	Brown–green (114)	Brown (95)	Dark brown (34)	–	–	–	–	–
Canopy density	Low (5)	Moderate (97)	High (141)	–	–	–	–	–
Branching	Low (8)	Moderate (128)	High (107)	–	–	–	–	–
Branch density	–	Moderate (105)	High (138)	–	–	–	–	–
Branch flexibility	Low (11)	Moderate (77)	High (155)	–	–	–	–	–
Skin color of perennial branch	Light green (20)	Brown–green (72)	Light brown (37)	Brown (114)	–	–	–	–
Skin color of current branch	White–green (23)	Light green (207)	Green (13)	–	–	–	–	–
Leaf density	–	Moderate (68)	High (175)	–	–	–	–	–
Leaf upper surface color	Light green (26)	Green (152)	dark green (65)	–	–	–	–	–
Transparency of leaf upper color	Transparent (14)	Relatively transparent (73)	Matt (156)	–	–	–	–	–
Leaf lower surface color	Silver–green (181)	Silver (62)	–	–	–	–	–	–
Leaf shape	Obovate (5)	Elliptic (46)	Elliptic–Lanceolate (152)	Lanceolate (40)	–	–	–	–
Leaf apex shape	Mucronate (26)	Cuspidate (87)	Acuminate (130)	–	–	–	–	–
Leaf base shape	Cuneate (176)	Acute (67)		–	–	–	–	–
Ripening date	Late‐August (30)	Early‐September (40)	Mid‐September (46)	Late–September (92)	Early–October (35)	–	–	–
Fruit density	Low (53)	Moderate (107)	High (83)	–	–	–	–	–
Fruit shape	Spherical (45)	Ovoid (106)	Elongated 92)	–	–	–	–	–
Fruit Symmetry	Symmetric (85)	Slightly asymmetric (88)	Asymmetric (70)	–	–	–	–	–
Fruit apex shape	Pointed (138)	Rounded (105)	‐	–	–	–	–	–
Fruit base shape	Truncate (149)	Rounded (94)	‐	–	–	–	–	–
Fruit nipple shape	Absent (118)	Tenuous (83)	Obvious (42)	–	–	–	–	–
Fruit color	Light green (42)	Green (50)	Green–purple (12)	Purple–green (28)	Purple–green (30)	Purple (3)	Dark purple (57)	Black (21)
Lenticel on fruit skin	Few and large (48)	Few and small (37)	Many and small (158)	–	–	–	–	–
Fruit flesh firmness	Low (24)	Moderate (90)	High (129)	–	–	–	–	–
Stone shape	Spherical (20)	Ovoid (118)	Elliptic (65)	Elongated (40)	–	–	–	–
Stone Symmetry	Symmetric (88)	Slightly asymmetric (109)	Asymmetric (46)	–	–	–	–	–
Stone apex shape	Pointed (195)	Rounded (48)	‐	–	–	–	–	–
Stone base shape	Truncate (55)	Pointed (100)	Rounded (88)	–	–	–	–	–
Stone color	Cream (41)	Light brown (130)	Brown (72)	–	–	–	–	–
Stone surface	Smooth (64)	Rugose (116)	Scabrous (63)	–	–	–	–	–
Groove number on stone	Low (91)	Moderate (146)	High (6)	–	–	–	–	–

### 2.3 Statistical analysis

Analysis of variance (ANOVA) was performed to evaluate the variation among cultivars based on the traits measured using SAS software (SAS Institute, 1990). Principal component analysis (PCA) was used to investigate the relationship between cultivars and determine the main traits useful in cultivars segregation using SPSS software. Hierarchical cluster analysis (HCA) was performed using Ward's method and Euclidean coefficient using PAST software (Hammer et al., 2001). The first and second principal components (PC1/PC2) were used to create a scatter plot with PAST software. Also, independent traits affecting the fruit weight as a dependent trait were determined through multiple regression analysis (MRA) using the “linear stepwise” method with SPSS software.

## 3 RESULTS AND DISCUSSION

There were significant differences among the cultivars studied based on the characters recorded. Mean of fruit number in inflorescence exhibited the highest CV (68.92%) and followed by fruit color (66.24%), fruit nipple shape (63.33%), and trunk color (60.60%), while the lowest CVs were related to petiole diameter (15.28%), fruit length (18.16%), stone diameter (19.40%), and stone length (19.47%). Overall, the CV was more than 20.00% in 46 of 50 characters measured. Lazovic and Adakalic (2020) studied an olive germplasm from Montenegro and reported that the CV for all the measured characters was lower than 20.00%.

Tree growth habit was drooping in 86, spreading in 99, and erect in 58 cultivars. Tree growth vigor, tree height, trunk diameter, and branching were predominantly moderate and then high. Leaf density was moderate in 68 and high in 175 cultivars (Table 2). Leaf shape showed high variation, including obovate (5 cultivars), elliptic (46), elliptic–lanceolate (152), and lanceolate (40). Leaf length ranged from 27.07 to 78.54 mm, leaf width varied from 5.42 to 23.06 mm, the range of petiole length was from 1.79 to 9.56 mm, and petiole diameter varied from 0.50 to 1.53 mm (Table 1). Leaf length and width are important varietal characters and are used for cultivar identification. They are genetic characters which may differ from cultivar to cultivar under similar soil and environmental conditions (Singh et al., 1999).

**FIGURE 1:**
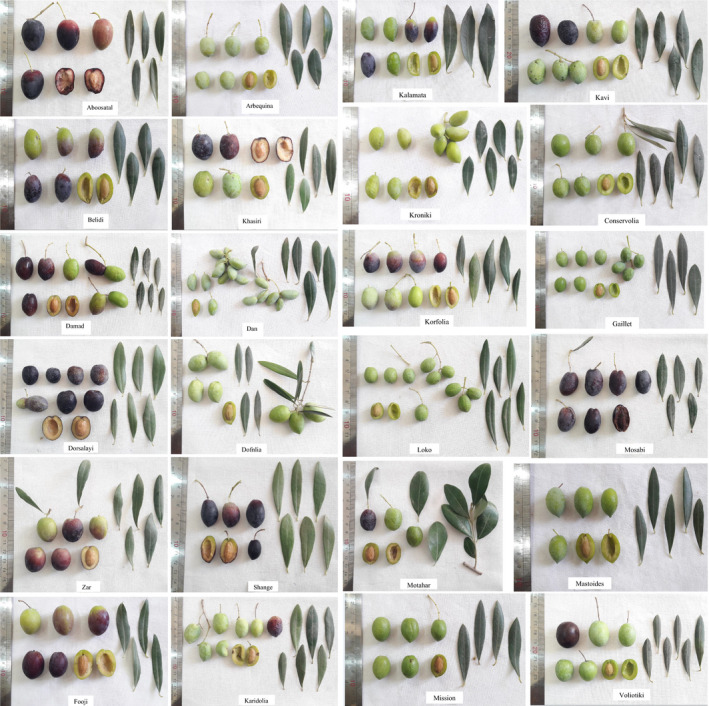
The pictures of leaves and fruits of olive cultivars studied

Ripening date ranged from late‐August to early‐October. Fruit density was low (53 cultivars), moderate (107), and high (83). The range of fruit number in an inflorescence was 1–10. Fruit length ranged from 13.04 to 33.72 mm, fruit diameter varied from 10.24 to 23.71 mm, fruit weighted from 0.97 to 9.61 g, and the range of fruit flesh thickness was 1.63–7.65 mm (Table 1). Fruit showed three shapes, including spherical (45), ovoid (106), and elongated (92) (Table 2). There was high variability in terms of fruit color, including light green (42 cultivars), green (50), green–purple (12), purple–green (28), purple–green (30), purple (3), dark purple (57), and black (21). Fruit flesh firmness was predominantly high (129 cultivars). The average of stone length, stone diameter, and stone weight was 16.35 mm, 8.22 mm, and 0.70 g, respectively. Fruit‐ and stone‐related traits are considered very efficient morphological characters in distinguishing among the cultivated olives (Lazovic et al., 2018; Peres et al., 2011; Rotondi et al., 2011). The pictures of leaves and fruits of the studied olives are shown in Figure 1.

Here, fruit weight was considered as a dependent variable and then the direct and indirect effects of each independent variable on this key trait were calculated using MRA (Table 3). The MRA showed that fruit weight was found to be associated with 18 characters. Fruit weight showed the highest positive standardized beta coefficient (*β*) value with stone weight (*β* = 0.61, *p* <.000). Thus, this key variable is one of the main traits accounting for fruit weight and should be considered in breeding programs.

**TABLE 3:** The traits associated with fruit weight in the olive cultivars as revealed using MRA and coefficients

Dependent character	Independent character	*r*	*r^2^*	*β*	*t* value	*p* value
Fruit weight	Fruit diameter	0.960 a	0.92	0.25	5.93	.00
	Stone weight	0.970 b	0.94	0.61	18.54	.00
	Flesh ratio to stone	0.980 c	0.97	0.27	13.05	.00
	Ripening date	0.981 d	0.97	0.07	5.53	.00
	Tree growth vigor	0.985 e	0.97	−0.05	−4.11	.00
	Fruit flesh thickness	0.986 f	0.97	0.12	4.19	.00
	Fruit density	0.987 g	0.98	−0.05	−4.83	.00
	Canopy density	0.988 hr	0.98	0.04	3.36	.00
	Leaf length	0.989 i	0.98	0.06	5.34	.00
	Stone apex shape	0.990 j	0.98	0.02	1.75	.08
	Stone length	0.991 k	0.98	0.08	4.89	.00
	Petiole length	0.992 L	0.98	−0.03	−2.67	.01
	Skin color of current branch	0.993 m	0.98	0.04	4.07	.00
	Mean of fruit number in inflorescence	0.994 *n*	0.98	0.05	3.10	.00
	Trunk diameter	0.995 o	0.98	−0.04	−3.21	.00
	Fruit nipple shape	0.996 p	0.98	−0.05	−3.46	.00
	Leaf apex shape	0.997 q	0.98	−0.03	−2.81	.01
	Fruit color	0.998 r	0.98	−0.03	−2.53	.01

The PCA was used to understand the relationships among the cultivars. The first 14 PCs explained 75.80% of the total variance (Table 4). The PCA has been used in the evaluation of olive germplasm (Bandelj et al., 2002; Cantini et al., 1999; Hannachi et al., 2008; Hosseini‐Mazinani et al., 2004; Lazovic & Adakalic, 2020; Lazovic et al., 2018; Strikic et al., 2009; Trentacoste & Puertas, 2011; Uylaser et al., 2008; Zaher et al., 2011). The first three PCs explained 31.46% of the total variance observed. The characters, including fruit length, fruit diameter, fruit weight, fruit flesh thickness, stone length, stone diameter, and stone weight, were positively correlated with PC1, explaining 14.72% of the total variance. Fruit size morphology is the product of complex genetic and environmental character (Strikic et al., 2009). Five characters, including fruit shape, fruit apex shape, fruit base shape, fruit nipple shape, and stone shape, were placed into the PC2, representing 10.74% of the total variance. The PC3 explained 6.00% of the total variance and showed positive correlations with tree growth vigor, tree height, and trunk diameter. Results obtained agreed with previous PCA of morphological characters in olive cultivars grown in different olive areas (Cantini et al., 1999; Lavee & Wonder, 2004; Lazovic et al., 2018; Ozkaya et al., 2006; Taamalli et al., 2006; Trentacoste et al., 2010; Zaher et al., 2011).

**TABLE 4:** Eigenvalues of the principal component axes from the PCA of the morphological characters in the studied olive cultivars

Character	Component													
	1	2	3	4	5	6	7	8	9	10	11	12	13	14
Tree growth habit	0.19	−0.02	0.48	−0.22	−0.16	0.09	−0.37	−0.15	−0.18	−0.13	−0.20	−0.02	0.21	0.37
Tree growth vigor	0.24	−0.03	0.63**	0.09	0.07	−0.14	0.01	0.14	−0.01	−0.21	0.36	−0.10	0.05	−0.01
Tree height	−0.10	−0.18	0.87**	0.08	0.07	0.05	0.00	0.14	−0.05	−0.02	−0.05	0.02	0.09	0.09
Trunk diameter	0.07	−0.05	0.77**	0.05	−0.02	0.15	0.08	0.19	0.11	0.14	0.05	−0.03	−0.18	−0.18
Trunk color	0.35	−0.24	0.42	0.01	−0.35	0.01	−0.23	−0.16	0.05	0.16	−0.20	−0.16	−0.41	0.14
Canopy density	0.06	0.08	0.13	0.83**	−0.03	0.05	−0.12	0.04	−0.05	−0.07	−0.06	−0.10	0.00	0.07
Branching	−0.12	0.20	0.03	0.44	−0.16	−0.20	0.25	−0.19	−0.09	−0.16	0.04	0.03	0.14	−0.51
Branch density	0.03	0.08	−0.04	0.85**	−0.03	−0.01	0.19	0.04	0.06	0.04	0.08	−0.03	0.13	−0.13
Branch flexibility	0.11	0.03	0.05	0.24	0.40	0.00	0.44	0.22	−0.07	−0.33	−0.26	−0.14	0.20	−0.11
Skin color of perennial branch	0.07	−0.12	−0.02	0.16	0.30	0.31	0.07	0.44	−0.12	−0.05	0.63**	0.03	0.10	−0.07
Skin color of current branch	−0.10	−0.18	0.00	−0.15	0.05	−0.10	0.05	0.01	−0.04	0.07	0.07	−0.01	−0.88**	0.06
Leaf density	−0.21	0.11	0.22	0.35	0.08	−0.09	0.44	−0.26	0.03	−0.26	0.21	0.08	−0.14	0.28
Leaf length	0.28	0.08	−0.04	−0.20	0.01	0.67**	0.09	−0.03	−0.01	−0.29	0.11	0.03	0.26	0.09
Leaf width	0.06	0.01	0.25	−0.07	−0.20	0.46	0.09	0.65**	−0.02	−0.28	0.14	−0.03	0.08	0.05
Petiole length	0.11	0.12	0.07	−0.07	0.06	0.68**	0.13	0.09	0.04	−0.19	0.13	0.16	0.02	0.27
Petiole diameter	0.06	−0.07	0.05	0.18	−0.02	0.76**	0.04	−0.02	−0.04	0.18	−0.06	0.00	−0.05	−0.17
Leaf upper surface color	−0.15	0.03	0.42	−0.05	−0.12	0.27	−0.02	0.09	−0.32	0.28	0.31	0.04	−0.10	−0.01
Transparency of leaf upper color	0.22	−0.12	0.07	−0.09	0.11	−0.13	−0.04	−0.17	0.12	0.75**	−0.01	−0.20	−0.08	0.08
Leaf lower surface color	−0.18	0.50	0.06	−0.03	−0.41	−0.18	−0.31	−0.26	0.19	0.08	−0.13	−0.07	0.33	−0.08
Leaf shape	0.10	0.08	−0.23	−0.09	0.20	0.12	−0.04	−0.83**	0.05	0.08	−0.02	0.06	0.10	0.04
Leaf apex shape	0.03	0.12	−0.02	−0.27	0.69**	−0.11	−0.03	−0.17	0.14	−0.07	0.08	0.09	−0.06	−0.12
Leaf base shape	0.01	−0.03	−0.02	−0.18	−0.74**	−0.05	0.03	0.03	0.14	−0.25	0.10	−0.05	0.06	0.02
Ripening date	−0.51	0.05	0.24	−0.06	0.27	−0.08	−0.22	−0.01	0.13	−0.04	0.10	0.40	0.02	−0.17
Fruit density	0.09	−0.30	−0.21	0.39	0.10	−0.09	−0.20	0.11	−0.09	0.42	0.12	0.21	0.00	0.05
Mean of fruit number in inflorescence	−0.50	0.16	0.06	0.32	0.12	0.03	0.06	−0.06	−0.43	0.08	−0.26	0.34	0.11	−0.16
Fruit stalk length	0.03	−0.07	0.39	−0.08	−0.10	−0.19	0.28	−0.15	0.16	0.00	0.03	0.31	0.07	0.03
Fruit stalk diameter	0.20	−0.21	0.07	0.15	−0.50	−0.05	−0.17	0.16	−0.03	−0.02	−0.34	0.13	−0.15	−0.11
Fruit shape	−0.15	0.83**	0.00	0.10	0.16	0.01	0.04	−0.02	−0.09	−0.04	−0.08	−0.09	0.11	0.22
Fruit symmetry	−0.24	0.41	−0.18	−0.13	0.21	0.17	0.25	0.30	−0.06	0.10	−0.08	0.20	−0.06	0.08
Fruit apex shape	0.02	−0.85**	0.12	−0.06	0.02	−0.10	0.09	0.10	−0.04	0.01	−0.09	−0.06	0.11	0.19
Fruit base shape	0.00	−0.77**	0.13	0.06	0.10	0.12	0.10	−0.13	0.13	−0.12	−0.18	0.10	−0.15	0.22
Fruit nipple shape	−0.06	0.79**	−0.13	0.01	0.07	0.04	0.01	−0.11	−0.09	−0.14	0.04	0.28	0.01	−0.13
Fruit length	0.85**	0.40	0.07	0.06	0.07	0.06	0.06	−0.01	0.14	0.01	−0.07	−0.04	0.08	0.14
Fruit diameter	0.93**	−0.16	0.04	0.00	−0.06	0.04	−0.02	−0.03	0.23	0.08	−0.02	−0.03	0.02	0.02
Fruit color	0.44	0.08	−0.09	−0.03	−0.43	−0.13	0.25	0.07	0.21	0.21	−0.04	−0.49	−0.09	0.10
Lenticel on fruit skin	−0.19	0.06	0.01	−0.07	0.01	−0.13	−0.83	−0.11	−0.01	0.06	0.03	−0.03	0.12	0.05
Fruit weight	0.94**	−0.08	0.02	0.04	0.01	0.07	0.01	−0.06	0.16	0.04	−0.06	−0.03	0.05	0.05
Fruit flesh firmness	−0.15	0.06	−0.07	−0.10	0.02	0.14	0.02	−0.01	−0.19	−0.06	0.05	0.78**	−0.02	0.01
Fruit flesh thickness	0.79**	−0.10	0.07	−0.01	−0.02	0.03	−0.04	−0.04	0.46	0.12	−0.07	−0.06	0.06	0.09
Stone shape	−0.19	0.83**	0.02	0.10	0.14	−0.04	0.04	0.03	0.10	−0.13	−0.24	−0.05	0.12	0.14
Stone symmetry	−0.15	0.43	0.12	0.05	0.10	0.02	0.29	0.47	0.04	0.27	−0.05	0.06	0.23	0.17
Stone apex shape	−0.18	−0.54	0.08	−0.04	0.11	−0.10	0.05	−0.09	−0.38	0.30	0.16	0.04	−0.03	0.30
Stone base shape	−0.35	−0.11	0.27	0.01	−0.06	0.04	0.00	−0.07	−0.13	0.06	0.64**	0.15	−0.17	0.04
Stone length	0.70**	0.57	0.04	0.07	0.14	0.06	0.05	0.04	0.08	−0.06	−0.15	−0.07	0.03	0.13
Stone diameter	0.90**	−0.26	0.03	−0.04	−0.09	0.08	0.04	0.02	−0.12	0.04	0.06	−0.02	−0.10	−0.06
Stone color	−0.28	0.07	0.04	−0.02	0.15	0.00	−0.03	0.01	−0.12	−0.07	−0.03	0.04	0.05	−0.60**
Stone surface	0.57	−0.32	0.03	−0.12	−0.03	0.16	−0.11	0.00	0.04	−0.08	−0.15	−0.23	−0.06	0.24
Groove number on stone	0.03	0.06	−0.06	0.14	0.02	−0.20	−0.50	−0.05	0.35	−0.05	−0.13	0.11	−0.18	−0.01
Stone weight	0.93**	−0.08	0.00	0.07	0.03	0.03	0.06	−0.02	−0.24	−0.02	0.03	0.02	0.05	−0.03
Flesh ratio to stone	0.26	−0.01	0.05	−0.04	−0.05	0.02	−0.05	−0.08	0.80**	0.13	−0.11	−0.22	0.07	0.14
Total	7.36	5.37	3.00	2.49	2.48	2.31	2.24	2.18	1.96	1.79	1.76	1.74	1.63	1.58
% of variance	14.72	10.74	6.00	4.99	4.95	4.61	4.48	4.36	3.92	3.58	3.53	3.49	3.26	3.17
Cumulative %	14.72	25.46	31.46	36.44	41.39	46.01	50.49	54.85	58.77	62.35	65.88	69.37	72.63	75.80

**Eigenvalues ≥0.60 are significant at the *p* ≤.01 level.

**FIGURE 2:**
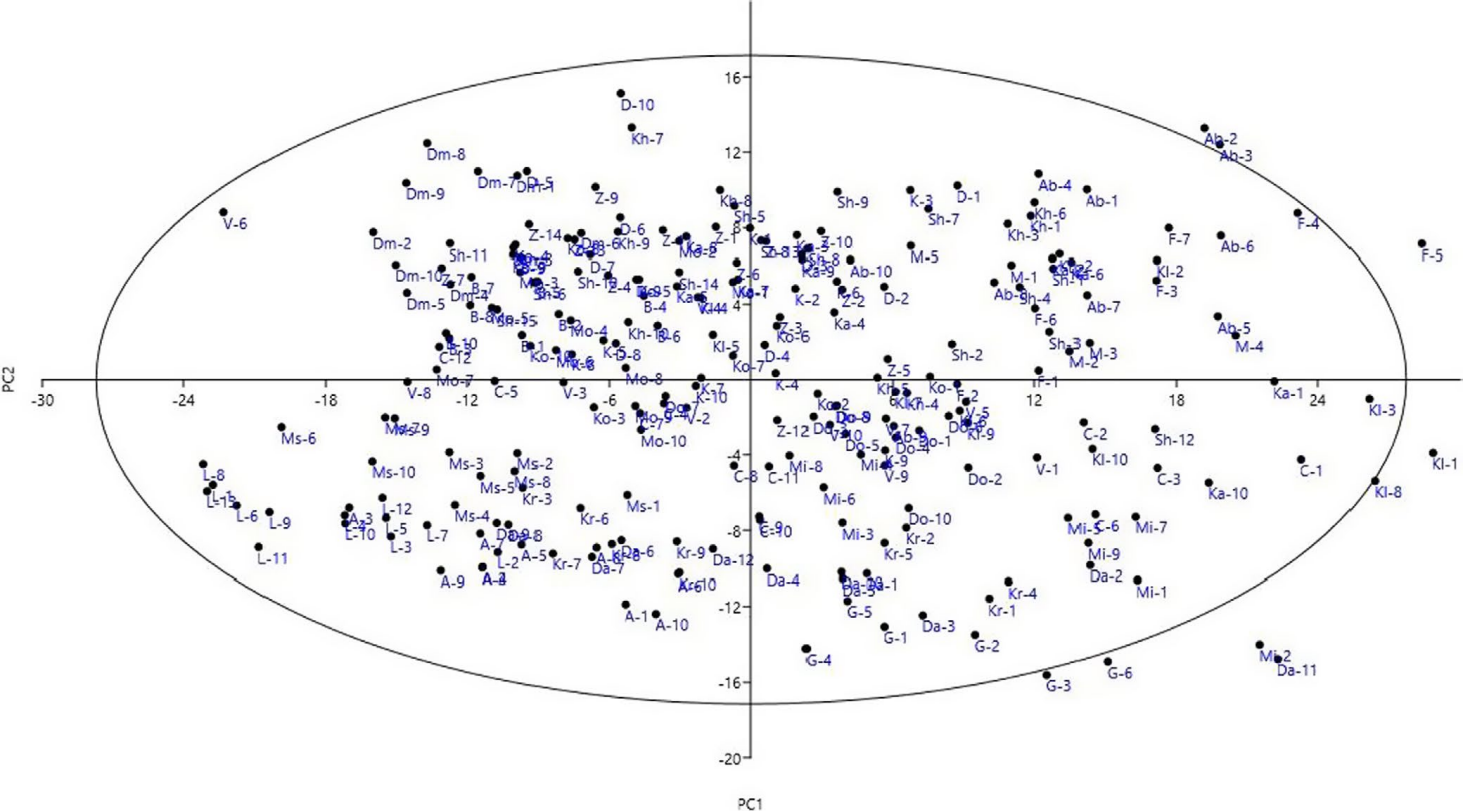
Scatter plot for the studied olive cultivars based on PC1/PC2. The symbols represent the olive cultivars in the plot, including Kavi (K), Khasiri (Kh), Dosalayi (D), Zard (Z), Shange (Sh), Motahar (M), Karidolia (Ka), Korfolia (Ko), Dan (Da), Mission (Mi), Conservolia (C), Gaillet (G), Fooji (F), Arbequina (A), Mastoides (Ms), Belidi (B), Kalamata (Kl), Kroniki (Kr), Damad (Dm), Loko (L), Aboosatal (Ab), Mosabi (Mo), Dofnlia (Do), and Voliotiki (V)

In addition, the scatter plot created based on the PC1 and PC2, accounted for 25.46% of the total variance (Figure 2), showed that the cultivars with close proximity were more similar in terms of effective traits in PC1 and PC2 and were placed in the same group. The scatter plot showed that residuals of the majority of cultivars bounce randomly around 0.00 line forming the horizontal band. This suggests that the variances in the error terms are equal and the relationship among the cultivars is linear. However, few outliers were observed among the cultivars evaluated, which might be due to their extreme values for particular traits.

**FIGURE 3:**
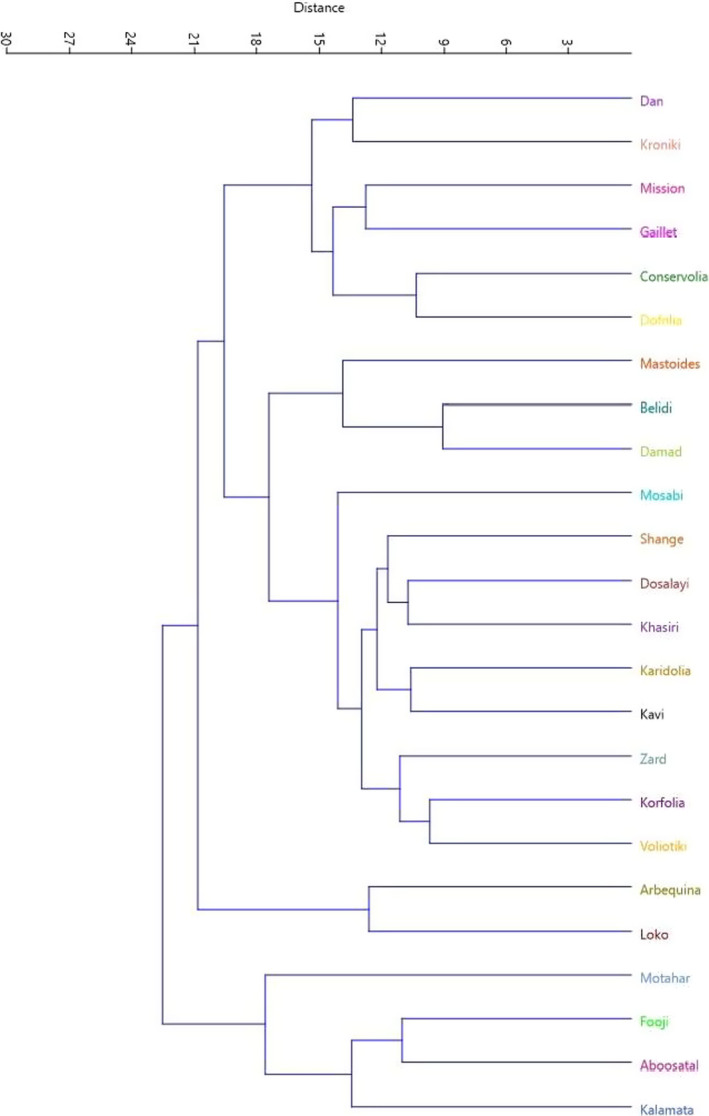
Ward cluster analysis of the studied olive cultivars based on the morphological and pomological traits by Euclidean distances

**FIGURE 4:**
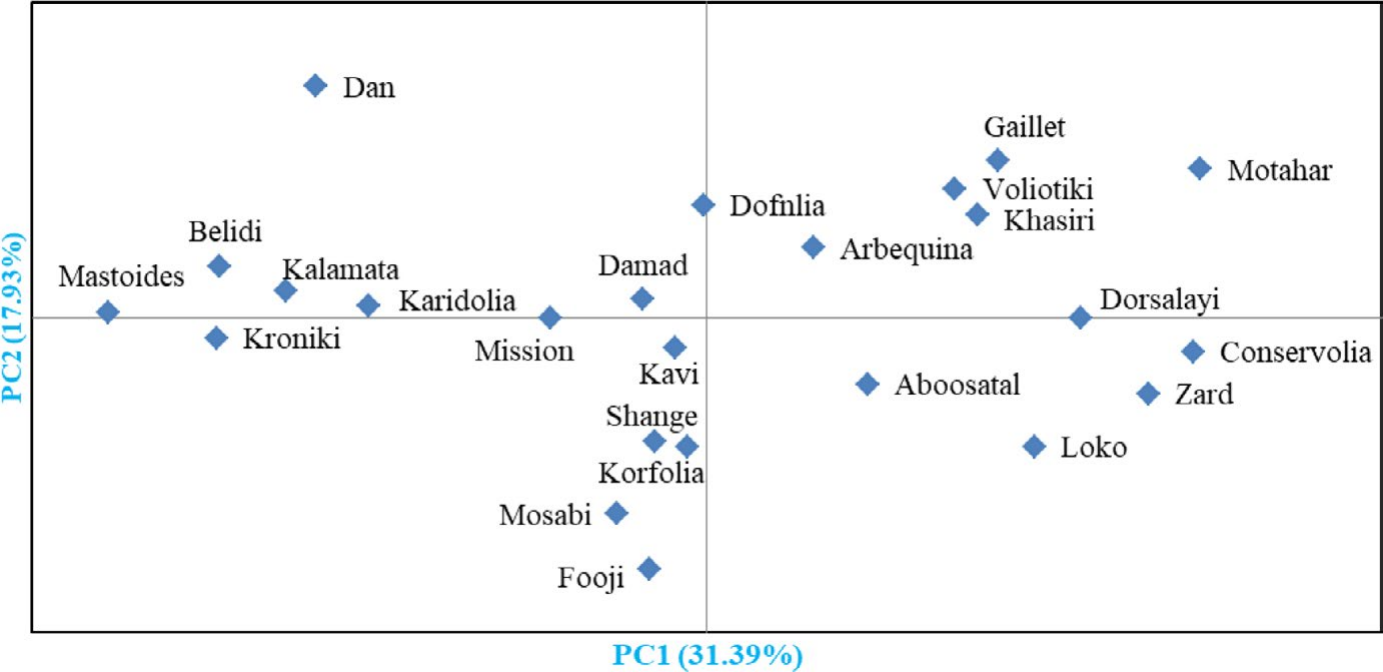
Biplot for the studied olive cultivars based on the morphological characters

Besides, the HCA performed based on the mean of replications with Euclidean distance and Ward method (Figure 3) grouped the cultivars into two major clusters. The first cluster (I) was divided into three subclusters. Subcluster I‐A consisted of six cultivars. Subcluster I‐B included 12 cultivars, while subcluster I‐C included 2 cultivars. The second cluster (II) included four cultivars. Furthermore, according to an analysis based on replications of cultivars (Figure 4), the studied cultivars were placed into four groups. The mean values of most important fruit traits for the studied olives are shown in Table 5.

**TABLE 5:** The mean value of most important fruit‐related traits of the olive cultivars studied

Cultivar	Fruit length (mm)	Fruit diameter (mm)	Fruit weight (g)	Fruit flesh thickness (mm)	Stone length (mm)	Stone diameter (mm)	Stone weight (g)
Kavi	22.99	16.67	3.90	4.27	16.74	8.89	0.85
Khasiri	23.80	18.99	5.21	4.45	15.90	10.70	1.18
Dosalayi	23.05	17.82	4.36	4.56	15.32	9.62	0.90
Zard	24.23	19.58	5.29	5.37	17.46	9.79	0.97
Shange5	24.65	16.33	3.53	4.38	18.27	8.41	0.67
Motahar	23.92	18.97	4.90	4.86	15.95	9.87	0.85
Karidolia	25.96	16.62	3.74	3.80	19.48	9.09	0.98
Korfolia	23.25	16.81	3.39	4.49	16.43	8.52	0.64
Dan	20.17	10.84	1.34	2.20	16.23	6.57	0.43
Mission	20.44	14.95	2.97	3.77	15.27	8.50	0.72
Conservolia	22.07	16.67	3.71	4.25	14.28	8.96	0.72
Gaillet	17.23	10.95	1.55	2.18	12.40	6.59	0.73
Fooji	28.01	21.04	6.62	5.70	19.81	9.85	1.12
Arbequina	15.34	11.80	1.37	2.59	11.90	6.79	0.35
Mastoides	21.44	12.98	1.86	3.54	15.73	6.03	0.36
Belidi	24.36	14.10	2.88	3.73	19.39	6.58	0.51
Kalamata	26.05	14.24	3.07	3.72	20.40	7.26	0.63
Kroniki	19.80	11.91	1.53	2.84	14.95	6.08	0.35
Damad	25.58	17.36	4.34	4.67	18.99	8.68	0.92
Loko	15.34	12.14	1.39	2.58	11.28	7.19	0.39
Aboosatal	28.10	19.47	6.25	5.85	20.78	8.84	0.87
Mosabi	19.39	14.59	2.42	3.65	13.64	7.61	0.49
Dofnlia	25.08	18.42	4.83	4.66	17.29	9.27	0.98
Voliotiki	21.07	16.58	3.27	4.88	14.25	8.04	0.53

The present study confirms previous studies in other countries on the importance of measuring morphological and pomological traits (Cantini et al., 1999; Lavee & Wonder, 2004; Lazovic et al., 2018; Ozkaya et al., 2006; Taamalli et al., 2006; Trentacoste et al., 2010; Zaher et al., 2011), which successfully classified cultivated olives. Furthermore, the evaluation of agronomic traits may be difficult since it may take as long as 10 years to reach reproductive maturity (Suarez, et al., 2011). Hannachi et al. (2008) found that there was a genetic basis in olive cultivars related to fruit size and probable fruit use.

## 4 CONCLUSION

The identification of olive cultivars and their area of origin are very important to expand cultivation of those commercial varieties with superior products that are best adapted to specific local environmental conditions. Differences in many of the morphological traits were observed across the cultivars. These sets of data were used to identify unique and desirable cultivars morphologically. Stable phenotypic traits were used to discriminate between use of fruit as well as cultivar origins (local or introduced). This research demonstrates that local olive cultivars have unique characteristics that differentiate them from imported cultivars. Thus, local cultivars provide novel genetic resources that should be conserved.

## CONFLICT OF INTEREST

The authors declare no conflict of interest.

## AUTHOR CONTRIBUTIONS

**Ali Khadivi:** Formal analysis (lead); Methodology (lead); Supervision (lead); Writing – review & editing (lead). **Farhad Mirheidari:** Investigation (equal). **Younes Moradi:** Investigation (equal). **Simin Paryan:** Investigation (equal).

## ETHICS STATEMENT

Research involving Human Participants and/or Animals: None.

## INFORMED CONSENT

None.

## Data Availability

The data that support the findings of this study are available from the corresponding author upon reasonable request.
